# Model-based clinical utility of pharmacogenetic testing and its potential population impact on the use of essential medicines in Zimbabwe

**DOI:** 10.3389/fphar.2026.1864897

**Published:** 2026-07-31

**Authors:** Tinashe Adrian Mazhindu, Zedias Chikwambi, Kevin V. Grimes, Margaret Z. Borok, Ntokozo Ndlovu, Collen Masimirembwa

**Affiliations:** 1 African Institute of Biomedical Sciences and Technology, Harare, Zimbabwe; 2 Department of Oncology, Medical Physics and Imaging Science, Faculty of Medicine and Health Sciences, University of Zimbabwe, Harare, Zimbabwe; 3 Department of Chemical and Systems Biology, Stanford University School of Medicine, Stanford, CA, United States; 4 Department of Internal Medicine, Faculty of Medicine and Health Sciences, University of Zimbabwe, Harare, Zimbabwe

**Keywords:** african genetic diversity, drug product information, genetics clinical utility, genomic medicine, pharmacogenetics, pharmacogenomic regulation, pharmacogenomics implementation

## Abstract

**Introduction:**

Pharmacogenomic-related adverse drug reactions and treatment failure contribute to global morbidity. Evidence for PGx-guided therapy in sub-Saharan Africa remains limited with few implementation studies. This study evaluated the potential clinical utility and projected population-level impact of pharmacogenomic testing in Zimbabwe.

**Methodology:**

A cross-sectional analysis used the Zimbabwe Essential Medicines List, DPWG guidelines, Zimbabwean genotype/phenotype data, and locally approved Summary of Product Characteristics. For the actionable gene–drug pairs identified, the number needed to genotype values were calculated from DPWG-defined absolute risk reduction and local genotype-phenotype frequencies. Potential clinical utility was assessed using the Clinical Implication Score framework and compared with Dutch population data.

**Results:**

Among 308 medicines screened, 30 (9.7%) contained actionable pharmacogenomic biomarkers, corresponding to 38 gene–drug pairs, all classified as vital or essential medicines. Lower NNG values were observed for UGT1A1–atazanavir (5), UGT1A1–irinotecan (9), CYP2B6–efavirenz (26), and CYP2C9–phenytoin (25), whereas 61% of gene–drug pairs had NNG values > 1,000 compared with 47% in the Dutch population. Overall, 81.6% of PGx recommendations were concordant across populations, while 21.1% were reclassified, primarily due to differences in local genetic profiles and the limited inclusion of PGx information in the Zimbabwean SmPCs, with 42% lacking PGx information compared with 24% in the Dutch dataset.

**Conclusion:**

Many essential medicines in Zimbabwe have actionable pharmacogenomic biomarkers, but gaps in local genomic guidance remain. Strengthening local evidence generation and PGx implementation is essential.

## Introduction

Adverse drug reactions (ADRs) and treatment failure (TF) remain major challenges in clinical therapeutics and represent an important cause of morbidity, mortality, and healthcare expenditure worldwide (Coleman and Pontefract, 2016). A proportion of observed inter-individual differences in drug efficacy and toxicity risk are attributable to inherited genetic variation affecting drug metabolism, transport, and pharmacodynamic pathways (Ahmed et al., 2016). Pharmacogenomics (PGx) aims to identify these genetic determinants of drug response and use this information to guide individualized clinical therapy, thereby improving treatment efficacy and reducing risk of adverse drug events (Evans and Johnson, 2001). Several organizations have created evidence-based pharmacogenomic guidelines to help clinicians select and dose medicines based on individual genotype as a component of personalized medicine (Abdullah-Koolmees et al., 2021; Kim et al., 2021). These collaborative groups evaluate gene–drug association studies and provide best-evidence clinical recommendations for dose adjustment, alternative medicine selection for indication, or enhanced patient monitoring based on genotype or assigned phenotype (Omran et al., 2026). Some of these testing and clinical action recommendations are also incorporated into regulatory guidance and drug labelling (Kim et al., 2021). Together, these resources represent a significant pool of the available knowledge base supporting the implementation of pharmacogenomics in clinical practice. Implementation of PGx-guided treatment has been observed to reduce adverse drug reactions by as much as 30% in a European setting (Van Der Wouden et al., 2020; Swen et al., 2023). It is important to note that this observation is strongly associated with the frequencies for the genotypes/phenotypes of concern in a given population and thus may differ across geographic and ethnic populations even when targeting the same gene-drug pairs for implementation (Nthontho et al., 2022).

Although pharmacogenomic prescribing guidance is increasingly accessible, its implementation varies significantly among global health systems (Cavallari et al., 2025). The Dutch Pharmacogenetics Working Group (DPWG) developed the Clinical Implication Score (CIS) to guide when pharmacogenetic testing is warranted, based on clinical effect, strength of evidence, number needed to genotype (NNG), and regulatory guidance. This framework allows for determining population-specific prioritisation of gene–drug interactions in clinical settings (Swen et al., 2018). In sub-Saharan Africa, where substantially greater genetic diversity exists, alongside distinct disease burdens, and differences in essential medicines lists, the clinical impact and projected population-level utility of pharmacogenetic testing may vary from that observed in the Dutch population (Kar et al., 2010; Laing et al., 2003; Rajman et al., 2017). Africa’s extensive genetic diversity presents challenges for identifying clinically relevant gene–drug interactions and establishing standardized PGx policies (Twesigomwe et al., 2025). Furthermore, the inclusion of CIS in the summary of product characteristics (SmPC) could also differ from one jurisdiction to the next based on National Regulatory Authority (NRA) capacity in medicine registration and licensing (WHO, 2025). Given the limited resources in sub-Saharan Africa, a scientifically sound method that minimizes the incidence of ADRs not only helping to improve health outcomes but can also guide effective use of scarce resources (Sukri et al., 2022).

Zimbabwe maintains a national Essential Medicines List (EDLIZ), which guides medicine procurement and prescribing in the public health sector (Organization WH, 2019). In the context of planning pharmacogenomic (PGx) implementation, evaluating the potential clinical and projected population-level impact of pre-emptive PGx testing requires systematic assessment of medicines in routine use, identification of those with actionable pharmacogenomic biomarkers, and estimation of key implementation metrics such as the NNG, representing the average number of patients who must be tested for a specific genetic variant to prevent one adverse drug reaction (ADR) or achieve an improved therapeutic outcome and the extent of PGx information included in Summary of Product Characteristics (SmPCs) (Tonk et al., 2017). This study aimed to evaluate the potential clinical utility and projected population impact of pharmacogenetic testing in Zimbabwe by focusing on essential medicines with known actionable pharmacogenomic biomarkers (MAPBs).

## Materials and methods

### Study design

This cross-sectional study evaluated the potential clinical utility of PGx implementation in Zimbabwe using the DPWG framework. The analysis integrated four data sources: (i) the national EDLIZ, (ii) Zimbabwean population pharmacogenomic genotype and phenotype frequencies (iii pharmacogenomic SmPC information contained in registered brands in Zimbabwe and (iv) clinical impact assessment using the DPWG CIS criteria and recommendations framework.

### Identification of medicines with actionable pharmacogenomic biomarkers

Medicines listed in the EDLIZ 9th edition (2025) were systematically extracted into a structured database together with their clinical indications. The study analysed systemic non-vaccine medicines only, excluding products classified as therapeutic foods, blood and blood derived products, dermatological agents, immunologicals, vaccines, ophthalmic preparations, dialysis solutions, electrolyte correction solutions, vitamin supplements, reproductive health products and other medical devices, local otorhinolaryngology and dental preparations. All medicines in the EDLIZ are categorized by level of availability and in the referral health system of the country and given a priority designation. Level of availability denotes: C- medicines required at primary healthcare centres and will be available at all levels of care; B- medicines to be found at secondary health centres, i.e., district hospitals and above; A- medicines prescribed in provincial and national central hospitals and S- medicines to be prescribed by specialist practitioners and/or require a diagnostic test before prescribing. Priority codes are used as a tool for purchase or availability prioritisation based on economic consideration. The codes are, V- vital, considered lifesaving, and unavailability would likely cause serious harm, thus the aim is to have constant availability; E- essential medicines are second level priority and without these, major discomfort or irreversible harm could occur and lastly, N- still necessary medicines but a lower priority than the V and E medicines. These local Zimbabwean designations are decided upon based on multiple factors including epidemiology, efficacy, and cost to the health system relative to other cost priorities.

The list of systemic medicines in EDLIZ was reviewed to identify those with established pre-emptive pharmacogenomic testing recommendations based on the DPWG guidelines, accessed via the KNMP pharmacogenomics database (accessed 10 January 2026). https://www.knmp.nl/dossiers/farmacogenetica/pharmacogenetics For each eligible gene–drug pair, the corresponding genotype/phenotype, DPWG-assigned clinical effect, level of evidence, NNG and PGx SmPC information criteria scores and overall CIS classification (based on the Dutch population) were extracted.

### Zimbabwe genotype/Phenotype frequencies

Genotype frequency data were derived from two previously conducted cross-sectional studies in Zimbabwe. The primary dataset included 522 healthy Black Zimbabwean volunteers aged 18–30 years, from whom venous blood samples were collected and genotyped using a 120-assay Open Array GenoPharm PGx panel, including CYP2D6 copy number variation analysis (Supplementary Table S1) (Mbavha et al., 2022). A secondary dataset (n = 90), generated using targeted Sanger sequencing, was used specifically to determine UGT1A1 genotype and phenotype frequencies. While the sample size was relatively limited, this represented the only available Zimbabwean dataset for UGT1A1 at the time of analysis. The resulting phenotype category frequencies were similar to those reported in independent studies of African ancestry populations, suggesting that the estimates are biologically plausible despite the wider uncertainty expected from a smaller sample (Wesonga et al., 2025). These genotype and phenotype frequencies were used to estimate the prevalence of actionable variants in the Zimbabwean population and to calculate the NNG using absolute risk reduction (ARR) estimates derived from DPWG data enabling the model-based implementation assessment. All ARR values used in calculating the NNG for the Zimbabwean population in this study were exactly the same as those determined by the DPWG in their CIS scoring. We utilized standardized allele function assignments and genotype-to-phenotype conversion tables based on PharmVar nomenclature. Phenotype groups were categorized into clinically relevant metabolizer groups (poor, intermediate, normal, rapid, or ultrarapid metabolizers). For pharmacogenes associated with immune-mediated adverse drug reactions (e.g., HLA alleles), the presence or absence of risk alleles was reported directly. The Genotype-to-phenotype translation details are provided in Supplementary Table S2.

### Assessment of pharmacogenomic information in SmPCs

For each identified essential medicine with an actionable PGx biomarker, the approved SmPC of registered brands with the Medicines Control Authority of Zimbabwe (MCAZ) were downloaded on 10 January 2026. (https://smpcs.mcaz.co.zw/). For each essential medicines with an actionable PGx biomarker, we recorded the total number of brands registered, the latest SmPC version was used, imported labels had the MCAZ approved version included. All approved SmPCs for registered brands underwent a systematic evaluation for the presence, clarity, clinical relevance, and extent of recommendations regarding pharmacogenomic testing based on the DPWG CIS framework (Supplementary Table S3). Wherever a PGx statement was present the statement was captured in its entirety and interpreted as such. Two independent investigators conducted the assessment independently, with any discrepancies resolved through consensus to ensure scoring accuracy. The highest-scoring SmPC was selected to reflect the maximum clinically available PGx guidance accessible to prescribers within the national regulatory environment.

### Overall clinical implication score

An overall CIS was determined for each gene–drug pair based on four criteria: (i) clinical effect, (ii) level of evidence, (iii) NNG, and (iv) pharmacogenomic information in SmPCs. In the scoring for Zimbabwe, the clinical effect and level of evidence scores were adopted directly from DPWG. For the NNG and SmPC scores, the calculated Zimbabwe-specific data were used. The combined CIS was used to classify gene–drug pairs into one of the three available recommendation categories: Potentially Beneficial, Beneficial, or Essential (Supplementary Table S3) (Swen et al., 2018). As shown, each criterion contributes a predefined number of points, which are summed to generate a total CIS ranging from 0 to 10. Using irinotecan and UGT1A1 as an example, the DPWG gave a total CIS of 8/10. First, reduced UGT1A1 activity (*28/*28 or poor metabolizer phenotype) is associated with potentially fatal irinotecan-induced toxicity (CTCAE grade 5), resulting in the maximum 2 points for clinical severity. Second, the association between UGT1A1 variants and severe neutropenia or diarrhoea is supported by more than three studies, yielding the maximum 3 points for level of evidence. Third, the estimated NNG in the Dutch population was 41, based on a 27% absolute reduction in grade 3–4 neutropenia among *28/*28 individuals and a population prevalence of 9%, corresponding to a score of 2 points for the NNG criterion (10 < NNG ≤100). Finally, the SmPC acknowledges increased haematological toxicity risk in *28/*28 patients but does not mandate genotyping, contributing 1 point. The summed score of 8/10 classified pre-emptive UGT1A1 genotyping before irinotecan therapy as clinically essential. For gene-drug pairs where no ARR is available from the DPWG, the framework still allows for scoring of the other criteria even when ARR-derived NNG values are unavailable.

### Statistical analysis

Allele and genotype frequencies were calculated and expressed as proportions with 95% confidence intervals (CI). Categorical distributions between the Zimbabwean and Dutch datasets were compared as descriptive percentages and absolute counts, where applicable. Inter-rater reliability for SmPC pharmacogenomic information scoring between the two independent investigators was assessed using Cohen’s Kappa coefficient along with overall percent agreement. The proportion of individuals with clinically actionable genotypes or phenotypes was determined for each selected medicine. Actionable phenotypes were defined as those for which DPWG guidelines recommend dose adjustment, alternative therapy, drug avoidance, or enhanced clinical monitoring. Number Needed to Genotype (NNG), defined as the number of individuals requiring testing to prevent one adverse drug event through genotype-guided therapy:

$$\text{NNG}=\frac{1}{\text{ARR}\times P_{\text{variant}}}$$

where:

- ARR represents the absolute risk reduction associated with genotype-guided therapy (sourced from DPWG data)All ARR values applied in the calculation of the NNG for the Zimbabwean population were the same as those used by the DPWG in the derivation of their CIS scores. If ARR was unavailable, NNG was not calculated and a default value of >1,000 (score = 0) was assigned.
-
$P_{\text{variant}}$ represents the prevalence of actionable genotypes or phenotypes in the Zimbabwean population. If no variant was observed, NNG was assigned as >1,000.

## Results

**FIGURE 1:**
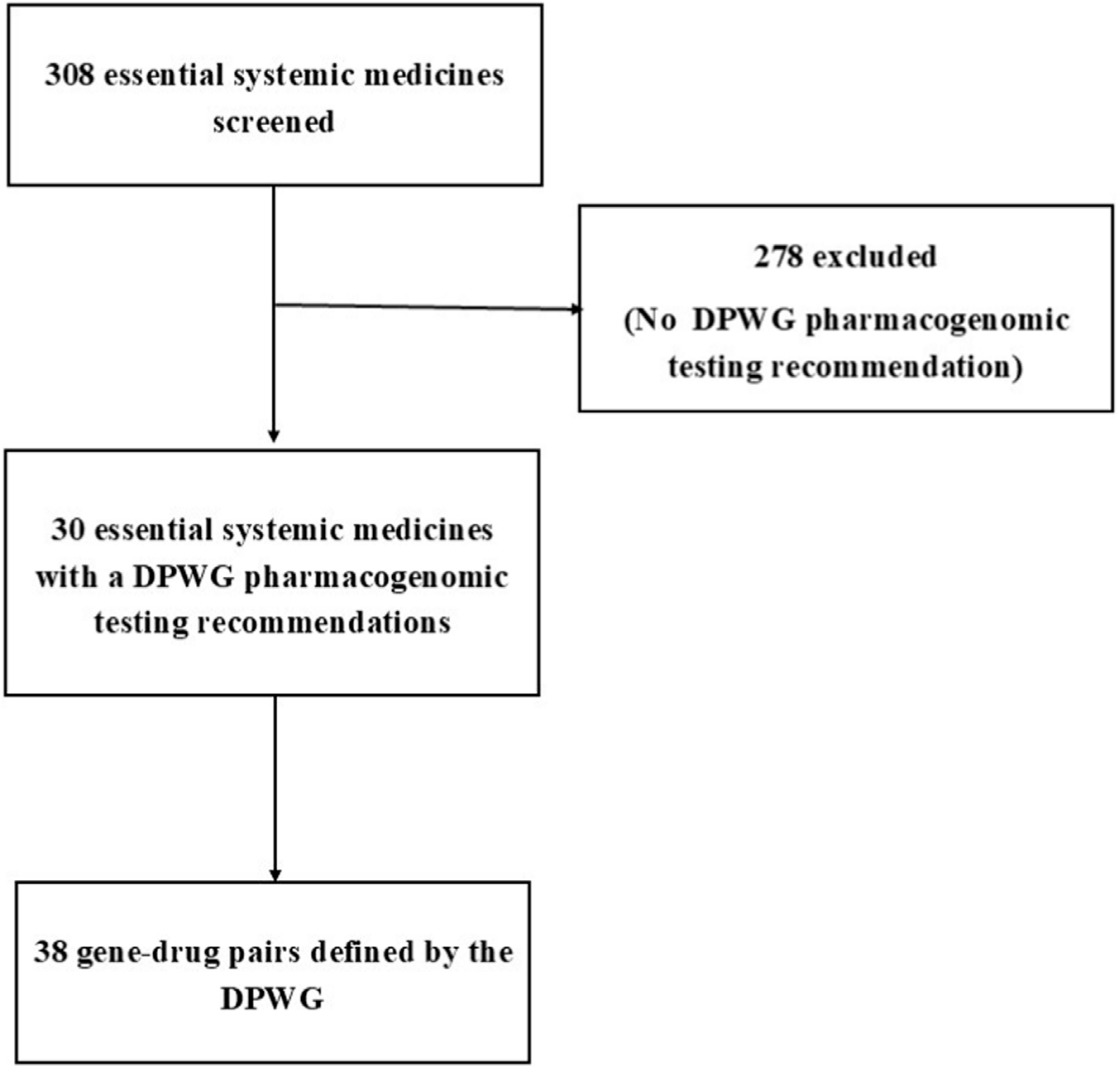
Flow chart showing how the gene-drug pairs were determined.

A total of 308 systemic medicines listed in the EDLIZ were screened for DPWG pharmacogenomic testing recommendations. From the review, 30 medicines (9.7%) with an actionable PGx biomarker were identified, translating to 38 gene-drug pairs with a testing recommendation (Figure 1).

**FIGURE 2:**
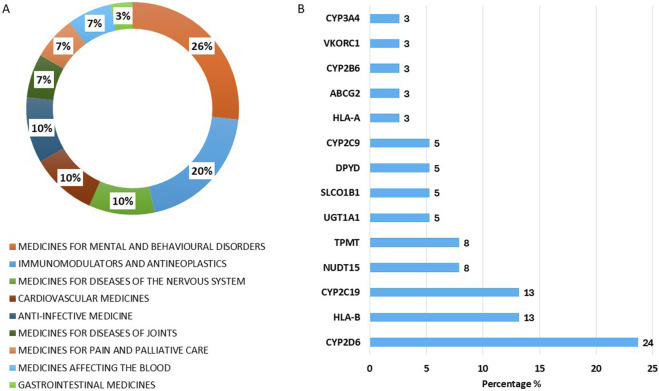
Distribution of PGx-Actionable Essential Medicines and Associated Pharmacogenes in Zimbabwe. **(A)** Breakdown by drug class for medicines with DPWG recommendations. **(B)** Frequency of specific pharmacogenes recommended for clinical testing.

All 30 medicines were categorized for use at secondary health system level or higher and classified as either vital or essential in the Zimbabwe health system (Table 1). Across the thirty medicines, the most represented drug class was medicines for mental and behavioural disorders (27%), followed by immunomodulators and antineoplastics (20%), medicines for diseases of the nervous system (10%), cardiovascular medicines (10%), and anti-infective medicines (10%) (Figure 2A). These drugs corresponded to fourteen different pharmacogenes, including genes involved in drug metabolism (CYP2D6, CYP2C19, CYP2C9, CYP2B6), immune-mediated hypersensitivity (HLA-B), drug transport (SLCO1B1, ABCG2), and toxicity susceptibility (DPYD, TPMT, NUDT15). The most frequent pharmacogenes were CYP2D6 (24%), HLA-B (13%), and CYP2C19 (13%) (Figure 2B).

**TABLE 1:** Essential medicines listed in Zimbabwe with a DPWG pharmacogenomic testing recommendations.

Drug	Gene	Gene variant/Phenotype	Drug class	Level of use	VEN
1. Abacavir	HLA-B	HLA-B*5701	Anti-infective medicinE	B	V
2. Allopurinol	HLA-B	HLA-B*58:01	Medicines for diseases of joints	B	E
	ABCG2	rs2231142	​	​	​
4. Amitriptyline	CYP2D6	UM/IM/PM	Medicines for mental and behavioural disorders	B	E
5. Atazanavir	UGT1A1	PM	Anti-infective medicine	B	V
6. Atorvastatin	SLCO1B1	rs4149056	Cardiovascular medicines	B	V
7. Azathioprine	NUDT15	IM/PM	Medicines for diseases of joints	S	E
	TPMT	IM/PM	​	​	​
9. Capecitabine	DPYD	AS 0,1, 1.5	Immunomodulators and antineoplastics	S	E
10. Carbamazepine	HLA-A	HLA-A*3101	Medicines for diseases of the nervous system	B	V
	HLA-B	HLA-B*1502	​	​	​
12. Citalopram	CYP2C19	IM/PM	Medicines for mental and behavioural disorders	B	V
13. Clopidogrel	CYP2C19	IM/PM	Medicines affecting the blood	B	E
14. Codeine	CYP2D6	UM/IM/PM	Medicines for pain and palliative care	B	E
15. Efavirenz	CYP2B6	IM/PM	Anti-infective medicine	B	V
16. Fluorouracil	DPYD	AS 0,1, 1.5	Immunomodulators and antineoplastics	S	E
17. Haloperidol	CYP2D6	UM/PM	Medicines for mental and behavioural disorders	B	V
18. Imipramine	CYP2D6	UM/IM/PM	Medicines for mental and behavioural disorders	B	E
	CYP2C19	PM	​	​	​
20. Irinotecan	UGT1A1	PM	Immunomodulators and antineoplastics	S	E
21. Lamotrigine	HLA-B	HLA-B*1502	Medicines for diseases of the nervous system	B	E
22. Mercaptopurine	NUDT15	IM/PM	Immunomodulators and antineoplastics	S	E
	TPMT	IM/PM	​	​	​
24. Metoprolol	CYP2D6	UM/IM/PM	Cardiovascular medicines	B	E
25. Omeprazole	CYP2C19	UM	Gastrointestinal medicines	B	E
26. Phenytoin	CYP2C9	IM/PM	Medicines for diseases of the nervous system	B	V
	HLA-B	HLA-B*1502	​	​	​
28. Quetiapine	CYP3A4	PM	Medicines for mental and behavioural disorders	S	E
29. Risperidone	CYP2D6	UM/PM	Medicines for mental and behavioural disorders	S	E
30. Rosuvastatin	SLCO1B1	rs4149056	Cardiovascular medicines	B	V
31. Sertraline	CYP2C19	PM	Medicines for mental and behavioural disorders	B	E
32. Tamoxifen	CYP2D6	IM/PM	Immunomodulators and antineoplastics	S	E
33. Thioguanine	NUDT15	IM/PM	Immunomodulators and antineoplastics	S	E
	TPMT	IM/PM	​	​	​
35. Tramadol	CYP2D6	UM	Medicines for pain and palliative care	S	E
36. Warfarin	CYP2C9	IM/PM	Medicines affecting the blood	B	V
	VKORC1	rs9923231	​	​	​
38. Zuclopenthixol	CYP2D6	IM/PM	Medicines for mental and behavioural disorders	B	V

Level of availability denotes: C- medicines required at primary healthcare centres and will be available at all levels of care; B- medicines to be found at secondary health centres, i.e., district hospitals and above; A-medicines prescribed at provincial and national central hospitals and S- medicines to be prescribed by specialist practitioners and/or require a diagnostic test before prescribing. The codes are, V- vital, considered lifesaving and unavailability would likely cause serious harm thus the aim is to have constant availability; E-essential medicines are second level priority and without these major discomfort or irreversible harm could occur and lastly, N-still necessary medicines but lower than the V and E medicines. PM-poor metabolizer, IM-intermediate metabolizer, NM-, normal metabolizer, UM-ultrarapid metabolizers, AS- activity score. rs- Reference SNP, cluster ID.

**FIGURE 3:**
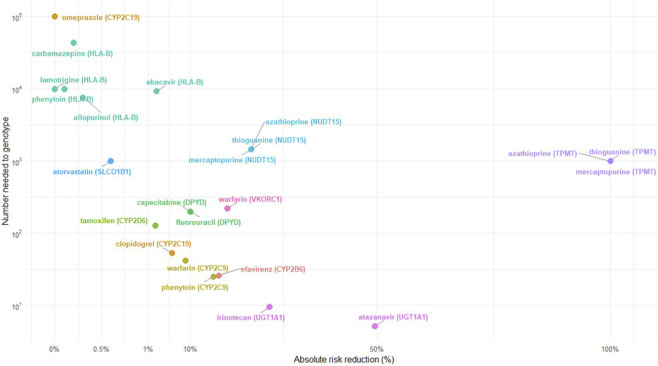
Nonlinear horizontal axis scatter plot of absolute risk reduction (ARR) versus number needed to genotype (NNG) for 23 drug–gene pairs. The x-axis shows ARR (in %) on a non-linear scale to enhance resolution at low values: the 0%–1% interval is expanded. The y-axis displays NNG on a scale to accommodate the wide range of values (from ∼5 to 100,000). Each data point represents a specific drug and the gene considered for testing; points are colored according to the gene. Data labels indicate the drug name followed by the gene in parentheses. The vertical dashed lines at 0%, 1%, and 100% are reference guides. The plot facilitates comparison of the clinical benefit (ARR) against the screening effort (NNG) for pharmacogenetic testing.

Application of the DPWG clinical implication framework to selected essential medicines in Zimbabwe demonstrated substantial variability in actionable pharmacogenomic variant/phenotype frequencies and subsequent NNG across drug–gene pairs. Based on the ARR used by the DPWG and genotype or phenotype frequencies in Zimbabwe observed from the pharmacogenomic datasets, the NNG values below were calculated as detailed in Table 2. Lower NNG profiles were observed for atazanavir–UGT1A1 (NNG 5), irinotecan–UGT1A1 (9), efavirenz–CYP2B6 (26), phenytoin–CYP2C9 (25), warfarin–CYP2C9 (42), tamoxifen–CYP2D6 (128), and DPYD-associated fluoropyrimidines (NNG 200). Only 5 gene-drug pairs have an NNG of <100. In contrast, 10 of the gene-drug pairs had an NNG <100 in the Dutch population. The other drug–gene pairs demonstrated much higher NNG values, with Zimbabwe having 23 pairs (61%) with NNG >1,000 compared to the Dutch population which has 18 pairs (47%). Thirty-eight drug–gene pairs were evaluated based on the ARR obtained for the Dutch population and of these 12 did not have a specified ARR or NNG and had a default NNG assignment of >1,000 (Figure 3). The stated reasons for this conclusion were that there were no observed severe clinical effects associated with the gene-drug pair (i.e., CTCAE E grade ≥ 3) which resulted additionally in the level of evidence score supporting associated clinical effect grade ≥3 events being assigned zero as well.

**TABLE 2:** Essential medicines in Zimbabwe, the DPWG PGx implementation absolute risk reduction (ARR) and calculated NNG for Dutch and Zimbabwean populations.

Drug/Gene	ARR used by DPWG	Dutch NNG	Frequency in Zimbabwe (%, 95% CI)	NNG Zimbabwe
Abacavir/HLA-B	2.7% (Mallal et al., 2008)	37	0.4% (0.1%–1.4%)	>1000
Allopurinol/HLA-B	0.3% (Ko et al., 2015)	333	4.4% (3.0%–6.5%)	>1000
Allopurinol/ABCG2	0.1% (Petru et al., 2016)	>1000	0.0% (0.0%–0.7%)	>1000
Amitriptyline/CYP2D6	NP	>1000	34.1% (30.2%–38.3%)	NC
Atazanavir/UGT1A1	49.6% (Lubomirov et al., 2011)	25	39.0% (35.0%–43.3%)	5
Atorvastatin/SLCO1B1	0.6 (KNMP, 2026)	>1000	0.0% (0.0%–0.7%)	>1000
Azathioprine/NUDT15	23.0% (Wang et al., 2018; Zhu et al., 2018; Chao et al., 2017)	20	0.3% (0.1%–1.4%)	1449
Azathioprine/TPMT	100.0% (Coenen et al., 2015)	384	0.1% (0.0%–1.1%)	1000
Capecitabine/DPYD	10.0% (Deenen et al., 2016)	163	5.0% (3.4%–7.2%)	200
Carbamazepine/HLA-A	0.1% (Yip et al., 2012; Grover and Kukreti, 2014; Bloch et al., 2014)	1818	NP	NC
Carbamazepine/HLA-B	0.2 (Yip et al., 2012; Grover and Kukreti, 2014; Bloch et al., 2014)	837	1.0% (0.4%–2.2%)	>1000
Citalopram/CYP2C19	NP	>1000	30.6% (26.8%–34.7%)	NC
Clopidogrel/CYP2C19	6.1% (Pan et al., 2017)	87	30.6% (26.8%–34.7%)	53
Codeine/CYP2D6	NP	>1000	2.9% (1.7%–4.7%)	NC
Efavirenz/CYP2B6	16.1% (Leger et al., 2016)	50	24.0% (20.5%–27.8%)	26
Fluorouracil/DPYD	10.0% (Deenen et al., 2016)	163	5.0% (3.4%–7.2%)	200
Haloperidol/CYP2D6	NP	>1000	3.5% (2.2%–5.4%)	NC
Imipramine/CYP2D6	NP	>1000	34.1% (30.2%–38.3%)	NC
Imipramine/CYP2C19	NP	>1000	2.1% (1.2%–3.7%)	NC
Irinotecan/UGT1A1	27.0% (Liu et al., 2017)	41	39.0% (35.0%–43.3%)	9
Lamotrigine/HLA-B	0.001 (Sabourirad et al., 2021; Deng et al., 2018)	>1000	1.0% (0.4%–2.2%)	>1000
Mercaptopurine/NUDT15	23.0% (Wang et al., 2018; Zhu et al., 2018; Chao et al., 2017)	20	0.3% (0.1%–1.4%)	1449
Mercaptopurine/TPMT	100.0% (Coenen et al., 2015)	384	0.1% (0.0%–1.1%)	1000
Metoprolol/CYP2D6	NP	>1000	2.9% (1.7%–4.7%)	NC
Omeprazole/CYP2C19	0.01%	>1000	2.9% (1.7%–4.7%)	>1000
Phenytoin/CYP2C9	15.0% (Fohner et al., 2019)	56	26.7% (23.0%–30.6%)	25
Phenytoin/HLA-B	0.1% (Cheung et al., 2013)	>1000	1.0% (0.4%–2.2%)	>1000
Quetiapine/CYP3A4	NP	>1000	0.0% (0.0%–0.7%)	NC
Risperidone/CYP2D6	NP	>1000	3.5% (2.2%–5.4%)	NC
Rosuvastatin/SLCO1B1	NP	>1000	0.0% (0.0%–0.7%)	NC
Sertraline/CYP2C19	NP	>1000	2.1% (1.2%–3.7%)	NC
Tamoxifen/CYP2D6	2.5% (Lum et al., 2013)	105	31.2% (27.4%–35.3%)	128
Thioguanine/NUDT15	23.0% (Wang et al., 2018; Zhu et al., 2018; Chao et al., 2017)	300	0.3% (0.1%–1.4%)	1449
Thioguanine/TPMT	100.0% (Coenen et al., 2015)	384	0.1% (0.0%–1.1%)	1000
Tramadol/CYP2D6	NP	>1000	2.9% (1.7%–4.7%)	NC
Warfarin/CYP2C9	9.0% (Yang et al., 2013)	43	26.7% (23.0%–30.6%)	42
Warfarin/VKORC1	9.0% (Yang et al., 2013)	38	2.5% (1.5%–4.2%)	222
Zuclopenthixol/CYP2D6	NP	>1000	31.2% (27.4%–35.3%)	NC

All ARR, values in the table were adopted from the DPWG. NNG-number needed to genotype; SmPC- summary of product characteristics; NP- ARR, not provided; NC- NNG, not calculated.

**FIGURE 4:**
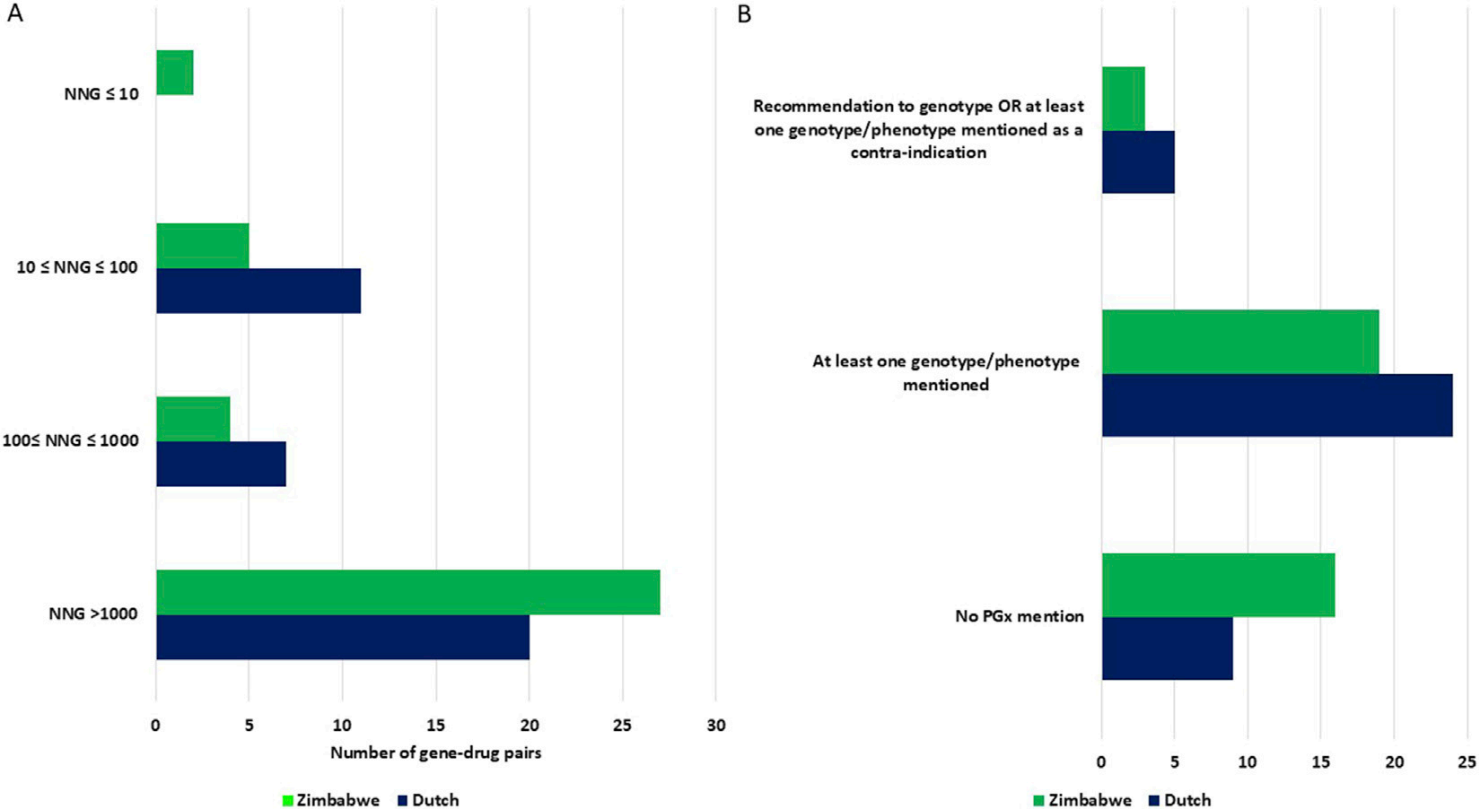
Comparative Analysis of PGx Metrics and Regulatory Guidance between Zimbabwe and the Netherlands. **(A)** Distribution of Number Needed to Genotype (NNG) values. **(B)** Comparison of pharmacogenomic information status within respective Summaries of Product Characteristics (SmPCs).

The full CIS scoring for the Zimbabwe and the established findings for the Dutch population is detailed in Table 3 below. For each drug varying number of registered brands were observed ranging from 1 to 16. The distribution of the NNG scores differed between the Dutch population and the Zimbabwean population. A greater proportion of gene–drug pairs in Zimbabwe are within the NNG category (>1000), accounting for 71% compared with 52.6% in the Dutch population. Moderate NNG ranges were less frequent in Zimbabwe, with 13% of gene–drug pairs in the 10–100 category compared with 28.9% in the Dutch dataset, and 11% versus 18.4% in the 100–1000 range, respectively. Low NNG values (≤10), were observed only in the Zimbabwean population (5%) and none observed for the Dutch population (Figure 4A).

**TABLE 3:** Clinical Implication Scoring for the Dutch and Zimbabwe incorporating Zimbabwe population genetic profile and registered drug SmPC PGx information.

Drug [number of brands registered]	Gene	Dutch					Zimbabwe				
		Clinical effect	Level of evidence	NNG	PGx SmPC	Total score	Clinical effect	Level of evidence	NNG	PGx SmPC	Total score
Abacavir [6]	HLA-B	++	+++	++	++	9	++	+++	​	++	7
Allopurinol [2]	HLA-B	++	+++	​	+	6	++	+++	​	+	6
Allopurinol [2]	ABCG2	+	​	​	​	1	+	​	​	​	1
Amitriptyline [4]	CYP2D6	​	​	​	+	1	​	​	​	+	1
Atazanavir [5]	UGT1A1	+	+++	++	​	6	+	+++	+++	​	7
Atorvastatin [8]	SLCO1B1	​	​	​	+	1	​	​	​	​	0
Azathioprine [1]	NUDT15	+	+++	++	+	7	+	+++	​	​	4
Azathioprine [1]	TPMT	++	+++	+	+	7	++	+++	​	​	5
Capecitabine [1]	DPYD	++	+++	+	++	8	++	+++	+	+	7
Carbamazepine [9]	HLA-A	+	+++	​	+	5	+	+++	​	+	5
Carbamazepine [9]	HLA-B	+	+++	+	++	7	+	+++	​	++	6
Citalopram [1]	CYP2C19	​	​	​	+	1	​	​	+	+	2
Clopidogrel [6]	CYP2C19	++	+++	++	+	8	++	+++	++	+	8
Codeine [16]	CYP2D6	++	​	​	++	4	++	​	​	​	2
Efavirenz [8]	CYP2B6	+	+++	++	+	7	+	+++	++	+	7
Fluorouracil [1]	DPYD	++	+++	+	++	8	++	+++	+	+	7
Haloperidol [1]	CYP2D6	​	​	​	+	1	​	​	​	+	1
Imipramine [1]	CYP2D6	​	​	​	​	0	​	​	​	​	0
Imipramine [1]	CYP2C19	​	​	​	​	0	​	​	​	​	0
Irinotecan [2]	UGT1A1	++	+++	++	+	8	++	+++	+++	+	9
Lamotrigine [2]	HLA-B	+	+++	​	​	4	+	+++	​	​	4
Mercaptopurine [1]	NUDT15	+	+++	++	+	7	+	+++	​	+	5
Mercaptopurine [1]	TPMT	++	+++	+	+	7	++	+++	​	​	5
Metoprolol [2]	CYP2D6	​	​	​	​	0	​	​	​	​	0
Omeprazole [5]	CYP2C19	+	​	​	+	2	+	​	​	+	2
Phenytoin [3]	CYP2C9	++	+++	++	+	8	++	+++	++	+	8
Phenytoin [3]	HLA-B	+	+++	​	+	5	+	+++	​	+	5
Quetiapine [4]	CYP3A4	​	​	​	​	0	​	​	​	​	0
Risperidone [1]	CYP2D6	​	​	​	​	0	​	​	​	​	0
Rosuvastatin [7]	SLCO1B1	​	​	​	+	1	​	​	​	+	1
Sertraline [1]	CYP2C19	​	​	​	+	1	​	​	​	+	1
Tamoxifen [2]	CYP2D6	++	+++	+	+	7	++	+++	++	​	7
Thioguanine [1]	NUDT15	+	+++	++	+	7	+	+++	​	++	6
Thioguanine [1]	TPMT	++	+++	+	+	7	++	+++	​	+	6
Tramadol [13]	CYP2D6	+	​	​	​	1	+	​	​	​	1
Warfarin [2]	CYP2C9	+	+	++	+	5	+	+	++	+	5
Warfarin [2]	VKORC1	+	+	++	+	5	+	+	+	+	4
Zuclopenthixol [1]	CYP2D6	​	​	​	+	1	​	​	​	​	0

NNG-number needed to genotype; SmPC- summary of product characteristics.

Analysis of SmPC pharmacogenomic information for registered essential medicines in Zimbabwe demonstrated 100% inter-investigator agreement across all 38 gene–drug pairs and due to the absence of variability in scoring between investigators, Cohen’s kappa statistic was therefore undefined. Analysis of pharmacogenomic information within SmPCs also demonstrated differences between the two settings. A higher proportion of medicines in Zimbabwe lacked any PGx information (42%) compared with the Dutch dataset (24%). Similarly, explicit recommendations for genotype-guided prescribing or contraindications based on PGx genotype or phenotype were more frequently observed in the Dutch dataset (13.2%) than in Zimbabwe (7.9%) (Figure 4B).

**FIGURE 5:**
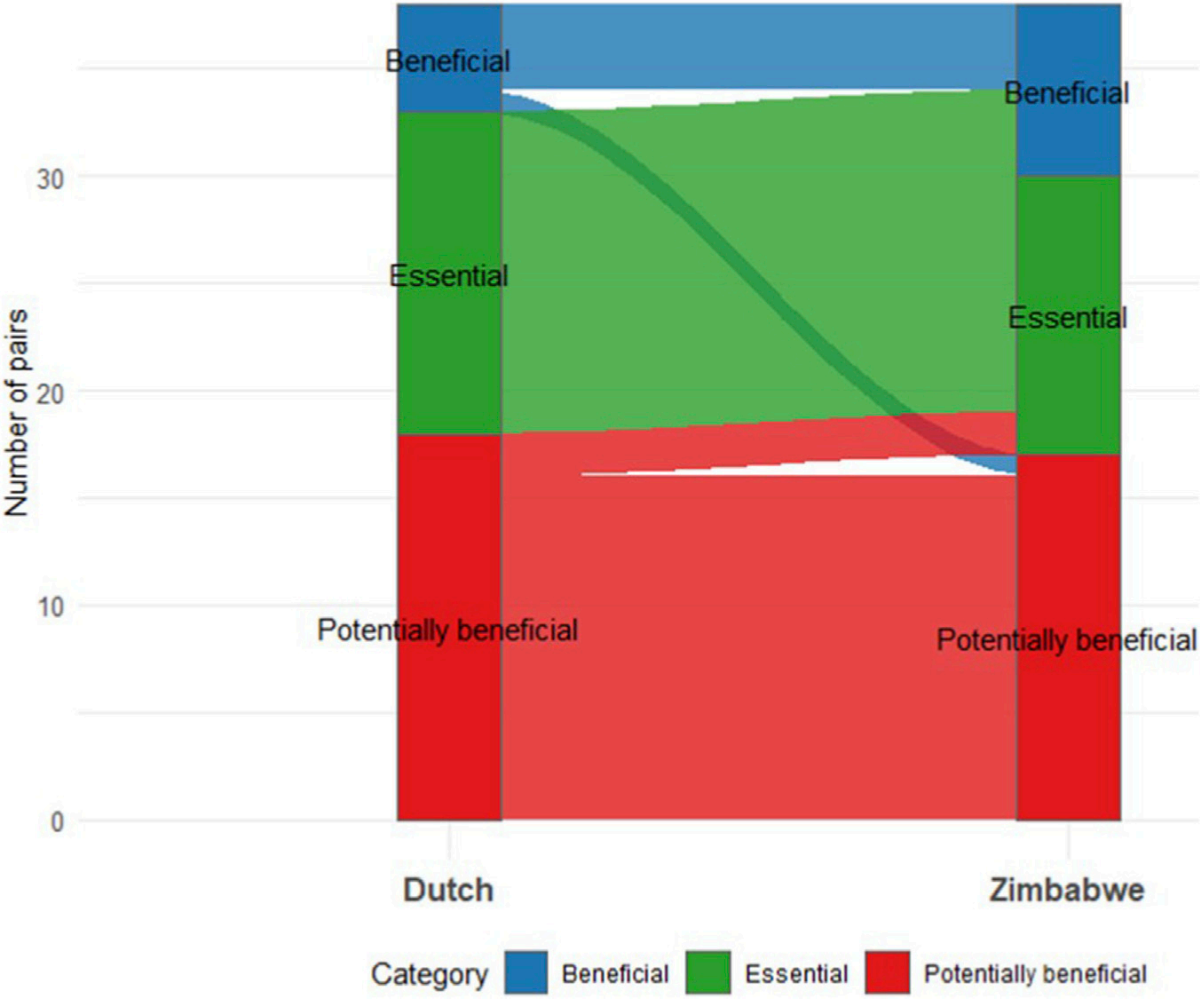
Sankey diagram comparing Dutch (left) and Zimbabwean (right) pharmacogenomic recommendation categories for 38 drug–gene pairs. Each flowing ribbon represents the number of drug–gene pairs transitioning from a Dutch recommendation category to a Zimbabwean category. The width of each ribbon is proportional to the count of pairs in that combination.

The PGx CIS total scores in Table 3 translate to a recommendation based on the DPWG categorisation described in Supplementary Table S2. Figure 5 compares the PGx recommendations between the Dutch and Zimbabwean populations and settings. There is a high degree of overall concordance, with 31/38 (81.6%) of drug–gene pairs showing no recommendation change. Consistent classification was observed for some high-impact and well-established PGx interactions, including HLA-B–abacavir, DPYD–capecitabine/fluorouracil, UGT1A1–irinotecan, and CYP2C9–phenytoin, which remained Essential in both populations. Similarly, drug–gene pairs such as warfarin (CYP2C9/VKORC1), carbamazepine (HLA-A), and lamotrigine (HLA-B) consistently remained within the Beneficial category. To a lesser extent and in contrast, 7/38 (18.4%) of drug–gene pairs had a change in recommendation, indicating population-specific genetic variation and/or differences in PGx information in approved SmPCs. Most changes reflected up-classification in the Zimbabwean population, atazanavir (UGT1A1), and efavirenz (CYP2B6), which was reclassified from Beneficial or Potentially beneficial to Essential. Conversely, drug–gene pairs that were down-classified, including codeine (CYP2D6) and thiopurine-related pairs (azathioprine and mercaptopurine with NUDT15 and TPMT), which shifted from Essential to Beneficial or from Beneficial to Potentially beneficial.

## Discussion

This study provides the first systematic evaluation of the potential clinical utility and population-level impact of pharmacogenomic (PGx) testing in Zimbabwe using the DPWG CIS framework. By integrating the Zimbabwean Essential Medicines List (EDLIZ) with local genotype-phenotype frequencies, and national medicine register data, we identified thirty essential medicines with actionable PGx biomarkers. Although the clinical utility of most gene–drug pairs was consistent between the Dutch and Zimbabwean populations, 18.4% required reclassification based on differences in local genetic profiles and regulatory information, underscoring the necessity for context-specific data for implementing precision medicine in some African populations and jurisdictions (Carrato, 2022). The estimated implementation impact of pharmacogenomics in clinical care offers an important opportunity for cost-effective pharmacoequity to address existing disparities in medicine development, safety, and efficacy among sub-Saharan African populations.

A key finding is the variability in the NNG, a metric that directly informs the cost-effectiveness of pre-emptive PGx testing (Sukri et al., 2022). Due to overall differences in NNG distributions clinically profound shifts occurred in high-burden therapeutic areas. The most striking examples were UGT1A1 with atazanavir and irinotecan, which exhibited higher NNG profiles in the Zimbabwean population studied due to the high prevalence of the UGT1A1 PM phenotypes (Wesonga et al., 2025). These findings have immediate public health implications: pre-emptive testing for these pairs could efficiently prevent severe toxicities, such as atazanavir-induced hyperbilirubinemia and irinotecan-induced neutropenia (Carrato, 2022; Shamu et al., 2024). Conversely, for other gene-drug pairs, the NNG exceeded 1,000. While this might suggest lower potential clinical utility, it more likely reflects a lack of characterised African-specific variants that contribute to adverse drug reactions (ADRs) or therapeutic failure (Dandara and Matimba, 2019).

The reclassification of PGx recommendations upgrading certain pairs to “essential” or “beneficial” demonstrates the importance of using local priority medicine lists. The upgrade for the efavirenz-CYP2B6 pair is a notable example. The high frequency of the CYP2B6 IM + PM observed in some African populations has been associated with elevated plasma concentrations, leading to central nervous system toxicity and treatment discontinuation (Masimirembwa et al., 2016; Park et al., 2023). Interpreted alongside efavirenz’s status as a “vital” medicine in Zimbabwe, our findings provide a data-driven rationale for prioritizing the PGx testing to ensure both safety and efficacy in national HIV programs (Leger et al., 2016).

A major challenge to PGx implementation identified in this study is the discrepancy in PGx information within SmPCs. While only 24% of Dutch SmPCs lacked PGx guidance, this figure rose to 42% in Zimbabwe. SmPCs are essential for providing evidence-based guidance to clinicians (Drelich et al., 2022). This information gap leaves healthcare providers without explicit information for dose modifications or toxicity monitoring, effectively stalling the adoption of PGx even where potential clinical utility is high. The comparatively lower representation of pharmacogenomic information within Zimbabwean SmPCs may reflect structural deficiencies in NRA systems, limited local understanding of the importance of pharmacogenomics in medicine safety, and the phenomenon of regulatory reliance in drug dossier approval (Owusu-Asante et al., 2026). In LMICs, it is likely that there is a slower speed of pharmacogenomic label updates, particularly if local regulatory authorities do not mandate stringent updates. Furthermore, dependence on reference agency approvals and imported regulatory documentation may contribute to delays in incorporating emerging pharmacogenomic evidence particularly that which is locally generated. Addressing this requires capacity building within NRAs and regulatory harmonisation through the newly formed African Medicines Agency (AMA) presents an opportunity to ensure that critical genomic safety information reaches the point of care across the region (Ncube et al., 2021). The incorporation of PGx data into patient-facing package inserts can empower patients, foster shared decision-making and potentially encouraging self-initiated testing (Haga et al., 2014).

These findings may help inform pharmacogenomic prioritization strategies in Zimbabwe and potentially other comparable sub-Saharan African settings. Our findings, though model-based, suggest a roadmap for integrating PGx into sub-Saharan health systems (Duong et al., 2020). First, identifying actionable essential medicines allows for targeted interrogation of pharmacovigilance data and the design of PCR panels tailored to the Zimbabwean population and certain sub-Saharan African populations (Kanji et al., 2023a). Second, health systems must bridge the “knowledge gap” by linking clinical outcomes, biobanking, and genomic data, allowing for calculation of local ARR rather than relying on extrapolating from non-African populations (Rajman et al., 2017; Mallal et al., 2008). Future research should focus on three priorities: expanding African genomic sequencing, conducting genotype–phenotype and cost-effectiveness studies in local populations, and addressing implementation barriers such as clinician knowledge, laboratory infrastructure, and patient acceptability (Lau-Min et al., 2022). These efforts could help policymakers and resource managers better direct genomic medicine deployment (Kim et al., 2021; Giri et al., 2019). This is aligned with Sustainable Development Goal target 3.8 on achieving universal health coverage, including access to quality essential healthcare services, safe and affordable medicines, and improved service coverage without imposing additional financial hardship on patients and vulnerable populations (SDG, 2026).

Implementation of pharmacogenomics in Zimbabwe will require consideration of existing cost constraints, limited availability of molecular diagnostic infrastructure, and the feasibility of centralized versus decentralized testing models. A pragmatic implementation strategy would prioritize gene–drug pairs based on a combination of clinical impact, severity of adverse outcomes, medicine importance within the EDLIZ, population frequency of actionable variants, and the NNG. Furthermore, recognizing the differences in toxicity-prevention PGx applications and that of diminished efficacy is needed. Using these criteria, high-priority candidates for early implementation include UGT1A1–irinotecan and DPYD–fluoropyrimidines due to their association with severe and potentially life-threatening toxicities, as well as CYP2B6–efavirenz because of the high burden of HIV treatment and the prevalence of actionable CYP2B6 variants in African populations. In the short term, targeted pharmacogenomic panels incorporating such high-evidence, high-impact gene–drug pairs may provide a practical and cost-effective approach to implementation in resource-limited settings. Integration of pharmacogenomic testing into established disease-specific programs, particularly HIV, oncology, and mental health services, could further facilitate scalable adoption by leveraging existing laboratory infrastructure, clinical workflows, and therapeutic monitoring systems.

This study has several limitations. First, the ARR values were derived from the DPWG and based primarily on European and Asian data. Several African-predominant PGx variants, such as CYP2D6 *17 and *29, remain incompletely characterised regarding their clinical impact (Kanji et al., 2023b; Mbavha et al., 2023; Mazhindu et al., 2025). This may influence CIS score interpretation. NNG estimates should be interpreted as strictly exploratory model-based estimates derived from published DPWG-associated ARR values rather than clinically validated outcomes in African populations. African populations due to differences in comorbidities, treatment adherence, co-medications, environmental exposures, and ancestry-specific modifiers may limit interpretation. This introduces uncertainty to directly applying these non-African clinical effect estimates to Africans for these various reasons. Furthermore, the DPWG committee’s methodology for ARR derivation, including the literature review process and other relevant parameters used to inform the selection of specific data sources, was not independently evaluated in this study. Second, the study population may not capture the full genetic diversity of Zimbabwe. Third, our analysis was restricted to EDLIZ and DPWG frameworks; other relevant interactions may have been excluded. In using the SmPC PGx information scoring, we adopted the highest-scoring SmPC which given the variability in labelling between brands may overestimate available PGx guidance in Zimbabwe. Other researchers who conduct a different methodology may come to a different score and conclusion. Finally, the CIS framework is subject to committee review and external factors like drug cost and availability, which were not evaluated here. It should also be noted that the genetic frequencies utilized may be influenced by substantial intra-African genetic diversity and genotype–phenotype variability, as highlighted by the Assessing Genetic Diversity in Africa (AGenDA) project. Consequently, the estimated phenotype frequencies, and the resultant NNG, and CIS scores may not be fully generalizable to all populations within Zimbabwe or across Africa (Ramsay et al., 2026).

The clinical applicability of pharmacogenomic testing in Zimbabwe is highly context-dependent. By applying the DPWG CIS model framework to local genetic and regulatory data, we identified a subset of essential medicines where PGx testing holds the greatest potential to improve patient outcomes. These findings highlight the importance of moving beyond imported strategies and investing in local data generation, regulatory capacity building, and health system preparedness to realize the benefits of personalized medicine in some African populations.

## Data Availability

The datasets presented in this study can be found in online repositories. The names of the repository/repositories and accession number(s) can be found below: Mendeley Data, V1, https://doi:10.17632/8c3r52859h.1.
