# Supplementary material for: Model-based clinical utility of pharmacogenetic testing and its potential population impact on the use of essential medicines in Zimbabwe
Source: Front Pharmacol. 2026 Jul 31;17:1864897. doi: 10.3389/fphar.2026.1864897 (PMC13473732; doi:10.3389/fphar.2026.1864897)
Supplement: Supplementary file 1 [file DataSheet1.pdf]

Supplementary Material

Supplementary Figures and Tables

Table S1: Gene variants tested for using the open array panel

Gene	Allele/variant tested
**Phase I ADME genes**	
**ADLH2**	Glu504Lys (rs671)
**CYP1A2**	*1C, *1D, *1F, *1K
**CYP2B6**	*4, *5, *6, *18
**CYP2C19**	*2, *3, *4, *7, *10, *17
**CYP2C9**	*2, *3, *4, *5, *6, *8, 11, C.1425A>T (rs1057911)
**CYP2D6**	*2, *2A, *4, *6, *7, *8, *9, *10, *11, *12, *14, *15, *17, *18, *19, *29, *31, *35, *41, *42, *44, *59, 4180G>C (rs1135840)
**CYP3A4**	*1B, *22
**CYP3A5**	*3, *6, *7
**CYP4F2**	*3
**DPYD**	*2A, *13, c.2846A>T, c.1129-5923C>G (HapB3), c.557A>A (rs115232898)
**Phase II ADME genes**	
**COMT**	Val158Met (rs4680)
**NAT2**	*5, *6, *7, *11, *12, *13, *14
**SULT4A1**	rs138097, rs138060
**TPMT**	*2, *3C, 3B, *4,
**UGT1A1**	*6 (rs4148323), -2936A>G (rs4124874)
**UGT1A6**	*4 (rs17863783)
**Transporters**	
**ABCB1**	c.1236T>C (rs1128503), c.2677T>G/A (rs2032582), c.3435C>T (rs1045642)
**ABCG2**	c.421C>A (rs2231142)
**SLC28A3**	L461L (rs7853758)
**SLC2A2**	T110I (rs5400)
**SLCO1B1**	*1B, *5, *15, *17, *21
**Drug Target**	
**VKROC1**	*2, *3F
**Other pharmacokinetic and pharmacodynamic genes**	

**ACE**	G2350A (rs4343)
**ADRA2A**	C1291G (rs1800544)
**AGT**	A-6G (rs5051)
**BDNF**	G196A (rs6265)
**CACNA1C-AS2.**	Rs1051375
**CDKN2B-AS1**	rs1333049, rs10757278 ACD risk
**CELF4**	rs1786814
**DRD2**	-241A>G (rs1799978)
**FABP2**	Ala54Thr (rs1799883)
**F2**	G20210 (rs1799963)
**F5**	R506Q (rs6025)
**G6PD**	376A>G (rs1050829)
**GRIK4**	83-10039T>C (rs1954787)
**GRIN2B**	rs2058878
**HLA-B**	*1502, *1502, *5701, *5801
**HTR2A**	rs7997012
**HTR2C**	-759C/T (rs3813929)
**IFNL3 (IL28B)**	(rs12979860)
**LPA**	I4399M (rs3798220), rs10455872
**MTHFR**	c.1298A>C (rs1801131), c.677C>T (rs1801133)
**NUDT15**	c.415C>T (rs116855232)
**OPRM1**	c.118A>G (rs1799971)
**RARG**	S427L (rs2229774)
**VEGFA**	-634C>G (rs2010963)
**Health, lifestyle, and performance genes**	
**ACTN3;**	R577X (rs1815739)
**TAS1R2**	Ile191Val (rs35874116)
**TMEM165;CLOCK**	3111C>T (rs1801260)

Table S1: Genotype-to-phenotype translation

Gene	Allele Function Assignment (per PharmVar/CPIC)	Activity Score (AS) Definition	Phenotype Conversion Rules	Key References
**CYP2D6**	• Normal function (1.0): *1, *2, *35<br>• Decreased function (0.5): *9, *17, *29, *41<br>• Decreased function (0.25): *10<br>• No function (0): *3, *4, *5, *6<br>• Increased (≥1.0 per copy): Duplications (*1xN, *2xN, *35xN)	Sum of both allele values:<br>AS = Value(allele1) + Value(allele2)	• PM: AS = 0<br>• IM: $0 < AS < 1.25$<br>• NM: $1.25 \leq AS \leq 2.25$<br>• UM: $AS > 2.25$	PharmVar: www.pharmvar.org/gene/CYP2D6<br>CPIC: https://cpicpgx.org/guidelines/<br>Consensus: Caudle et al. (2020), Clin Pharmacol Ther
**CYP2C19**	• Normal function (1.0): *1<br>• Increased function (1.5): *17<br>• No function (0): *2, *3, *4, *8<br>• Decreased function: rare alleles (*9, *10)	AS not uniformly applied; CPIC/DPWG provide direct diplotype-to-phenotype tables	• UM: *17/*17 or *1/*17 (some labs)<br>• RM: *1/*17 (some labs) or *17/*17<br>• NM: *1/*1<br>• IM: *1/*2, *1/*3, *2/*17<br>• PM: *2/*2, *2/*3, *3/*3	PharmVar: www.pharmvar.org/gene/CYP2C19<br>CPIC: https://cpicpgx.org/guidelines/
**CYP2C9**	• Normal function (1.0): *1<br>• Decreased function (0.5): *2<br>• No function (0): *3	AS = Value(allele1) + Value(allele2)	• NM: AS = 2.0 (*1/*1)<br>• IM: AS = 1.0–1.5 (*1/*2, *1/*3)<br>• PM: AS = 0–0.5 (*2/*2, *2/*3, *3/*3)	PharmVar: www.pharmvar.org/gene/CYP2C9<br>CPIC: https://cpicpgx.org/guidelines/
**CYP2B6**	• Normal function: *1<br>• Decreased function: *6, *9<br>• No function: *5, *7, *18	AS system less standardized; function assignment per CPIC tables	• NM: *1/*1<br>• IM: One decreased/no function allele<br>• PM: Two no function alleles	PharmVar: www.pharmvar.org/gene/CYP2B6<br>CPIC: https://cpicpgx.org/guidelines/
**CYP3A4**	• Normal function: *1<br>• Decreased function: *2, *3, *16, *22	No formal AS system; clinical guidance based on presence/absence of decreased function alleles	• NM: *1/*1<br>• IM/PM: Carrier of decreased function allele (dose reduction considered)	PharmVar: www.pharmvar.org/gene/CYP3A4<br>CPIC: https://cpicpgx.org/guidelines/
**TPMT**	• Normal function: *1<br>• No function: *2, *3A, *3B, *3C, *4	Phenotype based on number of no-function alleles (no AS system)	• NM: *1/*1 (2 normal alleles)<br>• IM: One no-function allele (e.g., *1/*2)<br>• PM: Two no-function alleles (e.g., *2/*2)	PharmVar: www.pharmvar.org/gene/TPMT<br>CPIC: https://cpicpgx.org/guidelines/
**NUDT15**	• Normal function: *1<br>• Decreased function: *2, *3, *4, *5, *9<br>• No function: *3 (homozygous)	Phenotype based on number of variant alleles	• NM: *1/*1<br>• IM: One variant allele (*1/*3, *1/*2)<br>• PM: Two variant alleles (*3/*3, *2/*2)	PharmVar: www.pharmvar.org/gene/NUDT15<br>CPIC: https://cpicpgx.org/guidelines/

**UGT1A1**	• Normal function: *1 (TA6/TA6)<br>• Decreased function: *28 (TA7/TA7), *6, *27	Phenotype based on combination of promoter repeats and coding variants	• NM: *1/*1<br>• IM: *1/*28 or *1/*6<br>• PM: *28/*28, *6/*6, or *28/*6	UGT Alleles Nomenclature: www.pharmacogenomics.pha.ulaval.ca<br>DPWG guideline (2023)
**SLCO1B1**	• Normal function: *1A, *1B<br>• Decreased function: *5, *15, *17 (c.521T>C)	No AS system; risk stratified by number of decreased function alleles	• Normal function: *1A/*1A<br>• Intermediate function: One decreased allele<br>• Low function: Two decreased alleles	PharmVar: www.pharmvar.org/gene/SLCO1B1<br>CPIC: https://cpicpgx.org/guidelines/
**DPYD**	• Normal function: *1<br>• Decreased function: c.2846A>T, c.1236G>A (HapB3)<br>• No function: c.1905+1G>A (*2A), c.1679T>G (*13)	CPIC uses gene-specific AS (DPYD activity score) based on summed variant function	• Normal (N): AS = 2 (two normal alleles)<br>• Intermediate (I): AS = 1–1.5 (one variant allele)<br>• Poor (P): AS = 0–0.5 (two variant alleles)	PharmVar: www.pharmvar.org/gene/DPYD<br>CPIC: https://cpicpgx.org/guidelines/
**VKORC1**	• High warfarin sensitivity (A): c.-1639G>A (3673G>A) – A allele<br>• Low warfarin sensitivity (G): G allele	No AS system; dose algorithm combines VKORC1 genotype with CYP2C9	• High sensitivity (A/A): requires lower warfarin dose<br>• Intermediate (G/A): intermediate warfarin dose<br>• Low sensitivity (G/G): requires higher warfarin dose	PharmGKB: www.pharmgkb.org<br>CPIC/DPWG warfarin dosing guidelines
**ABCG2**	• Normal function: wild-type<br>• Decreased/loss of function: c.421C>A (p.Q141K), c.1111_1112delAC	No AS system; activity classified as normal, reduced, or poor	• Normal: Standard dosing<br>• Reduced: Lower starting dose<br>• Poor: Alternative drug or significant dose reduction	PharmVar: www.pharmvar.org/gene/ABCG2<br>CPIC guidelines for statins/gout
**CYP3A5**	• Normal function: *1<br>• No function: *3, *6, *7	No formal AS; expressed vs. non-expressed phenotype	• Normal/Extensive (EM): *1/*1<br>• Intermediate (IM): *1/*3<br>• Poor (PM): *3/*3	PharmVar: www.pharmvar.org/gene/CYP3A5<br>CPIC tacrolimus guideline
**HLA-A & HLA-B**	• Risk alleles: Presence or absence of specific allele<br>• HLA-B: *57:01, *58:01, *15:02<br>• HLA-A: *31:01	No activity score system; binary (carrier vs. non-carrier)	• Positive: At risk for severe hypersensitivity reaction<br>• Negative: No increased risk	Allele frequency: dbMHC, Allele Frequency Net Database<br>CPIC HLA guidelines (abacavir, carbamazepine, allopurinol)

Table S3: Dutch Pharmacogenetics Working Group Clinical Implication Score Criteria and total score recommendations

SCORING	
Description	Assigned score
Clinical effects associated with gene/drug interaction	
CTCAE grade 3 or 4	(+)
CTCAE grade 5	(++)
Level of evidence supporting associated clinical effect	
One study with "level of evidence score" 3	(+)
At least two studies with "level of evidence score" 3	(++)
Three or more studies with "level of evidence score" 3	(+++)
Number needed to genotype (NNG) in the population under review	
100≤ NNG ≤ 1000	(+)
10 ≤ NNG ≤ 100	(++)
NNG ≤ 10	(+++)
Pharmacogenomics information in the SmPC	
At least one genotype/phenotype mentioned	(+)
Recommendation for genotype	(++)
At least one genotype/phenotype mentioned as a contra-indication in the corresponding section	(++)

INTERPRETATION		
Total score	Recommendation category	Description
0-2 +	Potentially beneficial	Pharmacogenomic testing for this gene-drug pair is potentially beneficial. Genotyping can be considered on an individual patient basis. If, however, the genotype is available, the DPWG recommends adhering to the gene-drug guideline
3-5 +	Beneficial	Pharmacogenomic testing for this gene-drug pair is beneficial. It is advised to consider genotyping the patient before (or directly after) drug therapy has been initiated to guide drug and dose selection
6-10 +	Essential	Pharmacogenomic testing for this gene-drug pair is essential for drug safety or efficacy. Genotyping must be performed before drug therapy has been initiated to guide drug and dose selection

CTCAE- common terminology criteria for adverse events; NNG- number needed to genotype; SmPC- Summary of Product Characteristics
